# Supplementary material for: Unconventional Hund metal in a weak itinerant ferromagnet
Source: Nat Commun. 2020 Jun 17;11:3076. doi: 10.1038/s41467-020-16868-4 (PMC7300033; doi:10.1038/s41467-020-16868-4)
Supplement: Supplementary file 1 — Supplementary Information [file 41467_2020_16868_MOESM1_ESM.pdf]

Supplementary Material
Unconventional Hund Metal in a Weak Itinerant Ferromagnet

Xiang Chen,[1] Igor Krivenko,[2] Matthew B. Stone,[3] Alexander I. Kolesnikov,[3] Thomas Wolf,[4] Dmitry Reznik,[5] Kevin S. Bedell,[6] Frank Lechermann,[7] and Stephen D. Wilson[1]

[1] *Materials Department, University of California, Santa Barbara, California 93106, USA*
[2] *Department of Physics, University of Michigan, Ann Arbor, Michigan 48109, USA*
[3] *Neutron Scattering Division, Oak Ridge National Laboratory, Oak Ridge, Tennessee 37831, USA*
[4] *Institute for Solid State Physics, Karlsruhe Institute of Technology, 76131 Karlsruhe, Germany*
[5] *Department of Physics, University of Colorado at Boulder, Boulder, Colorado 80309, USA*
[6] *Department of Physics, Boston College, Chestnut Hill, Massachusetts 02467, USA*
[7] *I. Institut für Theoretische Physik, Universität Hamburg, 20355 Hamburg, Germany*

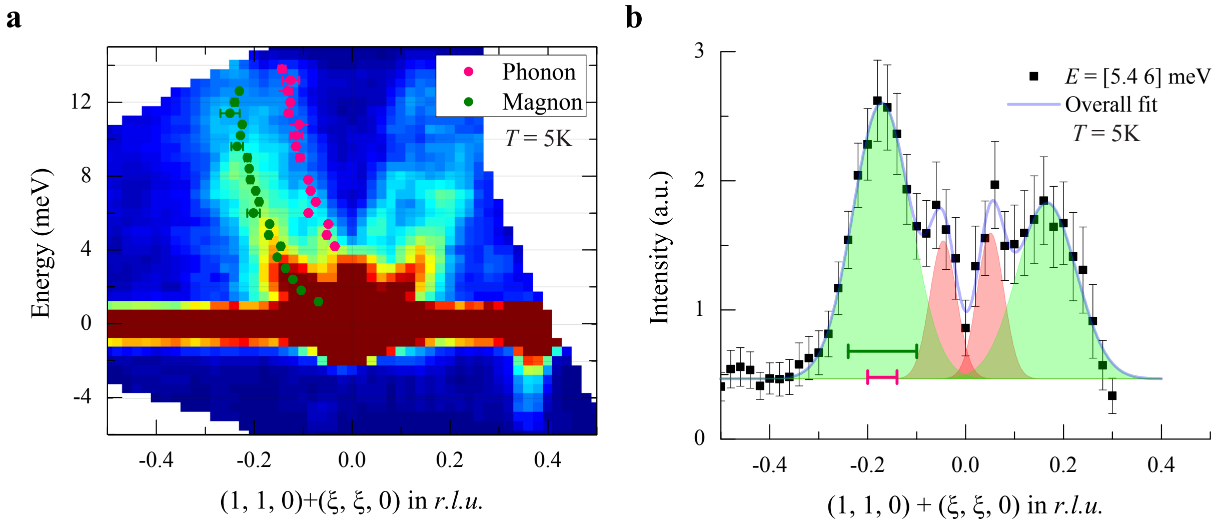

Supplementary Figure 1. (**a**) Representative energy-momentum (E - $\mathbf{Q}$) slice about the (1, 1, 0) zone center along (ξ, ξ, 0) direction, collected with incident energy $E_{\rm i}$ = 18 meV at T = 5 K (dE = 0.6 meV, $dq_{||}$ = 0.02 r.l.u., $dq_{\perp}$ = 0.06 r.l.u.). The scattered symbols are obtained from constant energy cuts of the E - $\mathbf{Q}$ slice, with one typical cut is shown in (**b**). (**b**) Constant-E cut in the energy range of 5.4 to 6 meV, fit with four Gaussians plus a constant background. The spin wave excitation peaks and acoustic phonon peaks are constraint to have the same peak widths, respectively. The horizontal bars display fitted FWHMs (pink bar for phonons and olive-green bar for spin waves). Note, while the left and right phonon peaks (pink) are almost identical, the magnetic excitation peaks have different intensities. This arises from the uncorrected magnetic form factor in the raw data plotted here. Error bars represent one standard deviation of the data.

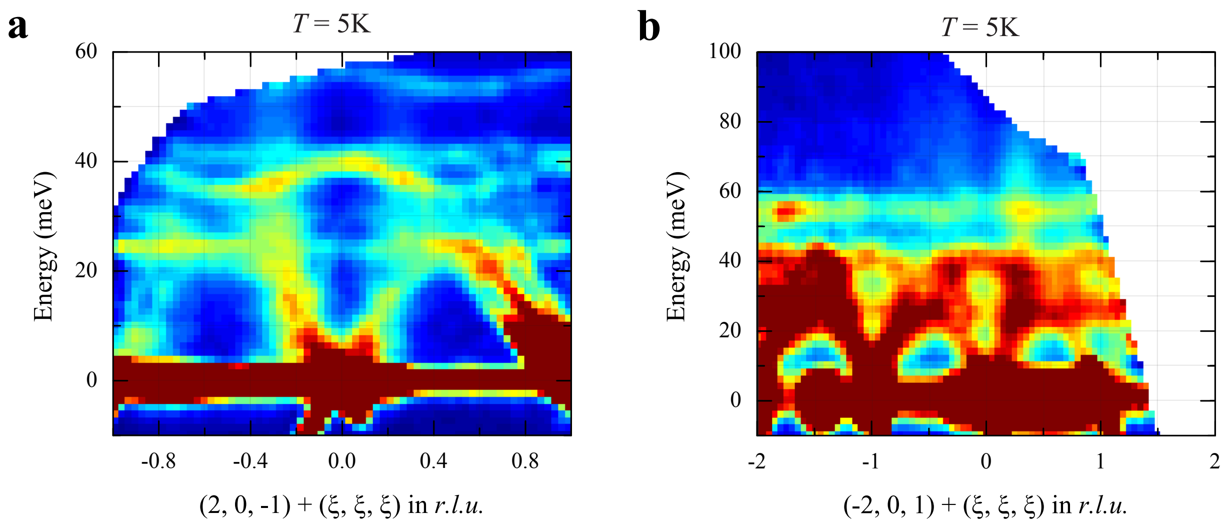

Supplementary Figure 2. Typical E - $\mathbf{Q}$ slices at T = 5 K. (**a**) E - $\mathbf{Q}$ slice collected with E_{i} = 70 meV (dE = 1.25 meV, $dq_{||}$ = 0.03 r.l.u., $dq_{\perp}$ = 0.1 r.l.u.), around the (2, 0, −1) zone center along high symmetry (ξ, ξ, ξ) direction. Prominent optical phonon bands exist in the energy range E=[20 45] and E=[50 60] meV, which leaves an optical phonon-free window between 45 and 50 meV. (**b**) E - $\mathbf{Q}$ slice collected with E_{i} = 150 meV (dE = 2.0 meV, $dq_{||}$ = 0.05 r.l.u., $dq_{\perp}$ = 0.15 r.l.u.), around the (−2, 0, 1) zone center along high symmetry (ξ, ξ, ξ) direction. The phonon cutoff energy is near 60 meV, which is apparent from the the highest optical phonon branch centered at ≈ 55 meV. The magnetic excitations above 60 meV are most apparent about the (−2, 0, 1) zone center. Error bars represent one standard deviation of the data.

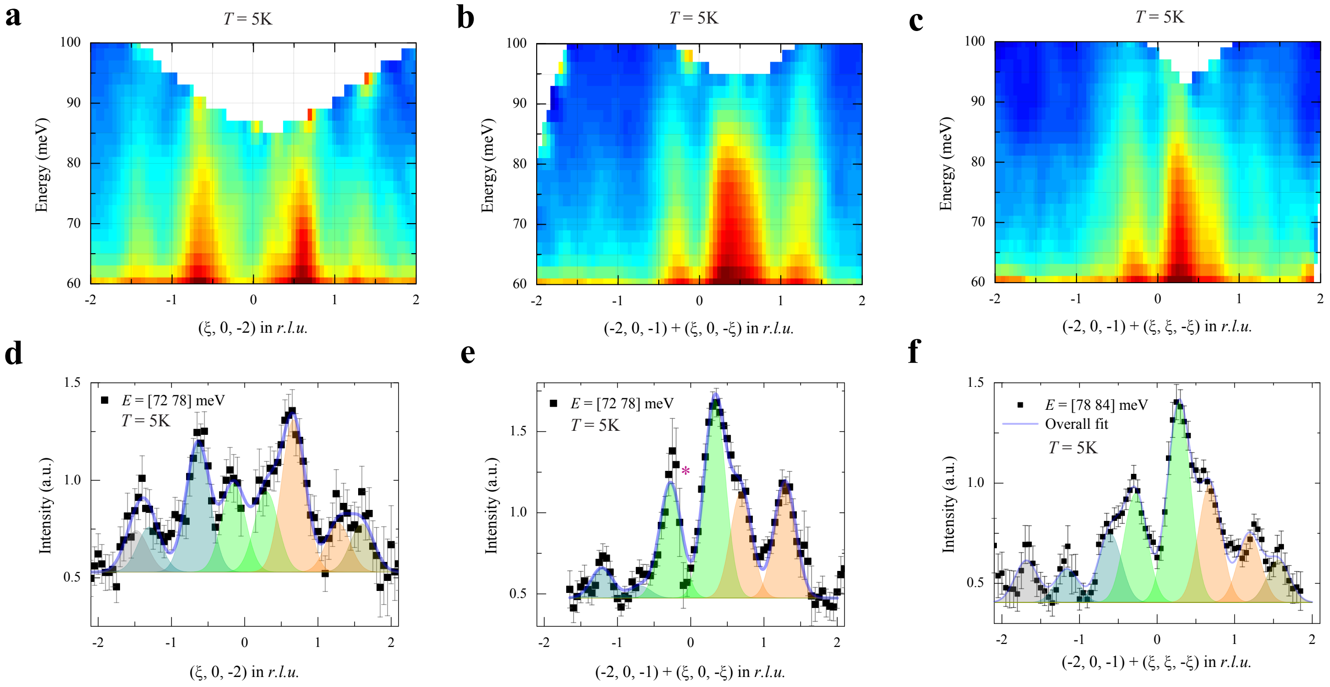

Supplementary Figure 3. Typical E - $\mathbf{Q}$ slices above the phonon cut-off energy along different high symmetry directions with $E_{\mathrm{i}} = 150$ meV at $T = 5$ K and corresponding representative constant-E cuts. (**a**) E - $\mathbf{Q}$ slice along (ξ, 0, 0) direction around the (0, 0, -2) zone. (**b-c**) E - $\mathbf{Q}$ slices collected around the (-2, 0, -1) zone along the (ξ, 0, $-\xi$) and (ξ, ξ, $-\xi$) directions, respectively. (**d-f**) Constant-E cuts, corresponding to the E - $\mathbf{Q}$ slices in **a-c**, respectively. The data are fit with Gaussians with a constant background. The shaded peaks with same color represent pairs of magnetic excitations emanating from the same zone. The different intensities between the pairs of peaks are mainly due to uncorrected magnetic form factor; while the detector gaps (magenta asterisk) may also influence the peak intensities. The E - $\mathbf{Q}$ slices in **a-c** are obtained with $dE = 2.0$ meV, $dq_{||} = 0.05$ r.l.u., $dq_{\perp} = 0.2$ r.l.u., and for constant E cuts in **d-f**, $dq_{\perp} = 0.1$ r.l.u.. Error bars represent one standard deviation of the data.

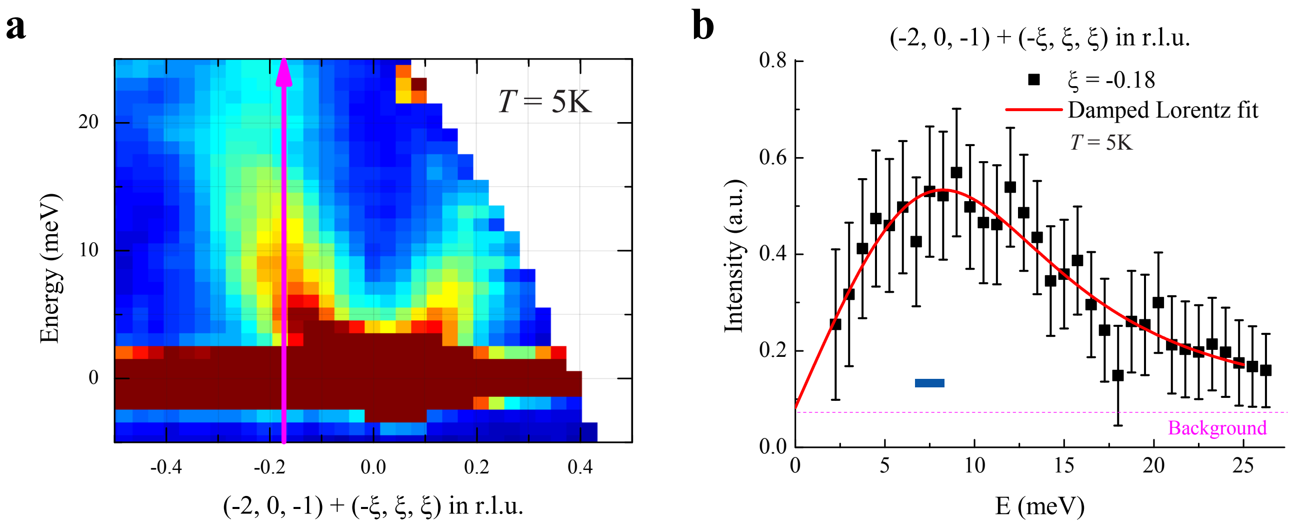

Supplementary Figure 4. Damping constant γ from a constant-**Q** cut. (**a**) E - **Q** slice along $(-\xi, \xi, \xi)$ direction around the $(-2, 0, -1)$ zone center, collected with $E_{\rm i} = 30$ meV at $T = 5$ K ($dE = 1.0$ meV, $dq_{||} = 0.03$ r.l.u., $dq_{\perp} = 0.075$ r.l.u.). (**b**) Energy dependence of the damped spin wave intensity at constant $\mathbf{Q} = (-1.82, -0.18, -1.18)$ r.l.u. (at $\xi = -0.18$ r.l.u.). The data is fit with a damped Lorentzian of the form $\frac{\gamma E}{(E^2 - E_0^2)^2 + (\gamma E)^2}$, where E_0 is the mode energy. The direction of constant-**Q** cut is indicated by the magenta arrow in **a**. The experimental energy resolution at the elastic scattering position is indicated by the blue horizontal bar in **b**. The background is estimated from the constant **Q** cut at the zone boundary $\xi = -0.5$ r.l.u.. Error bars represent one standard deviation of the data.

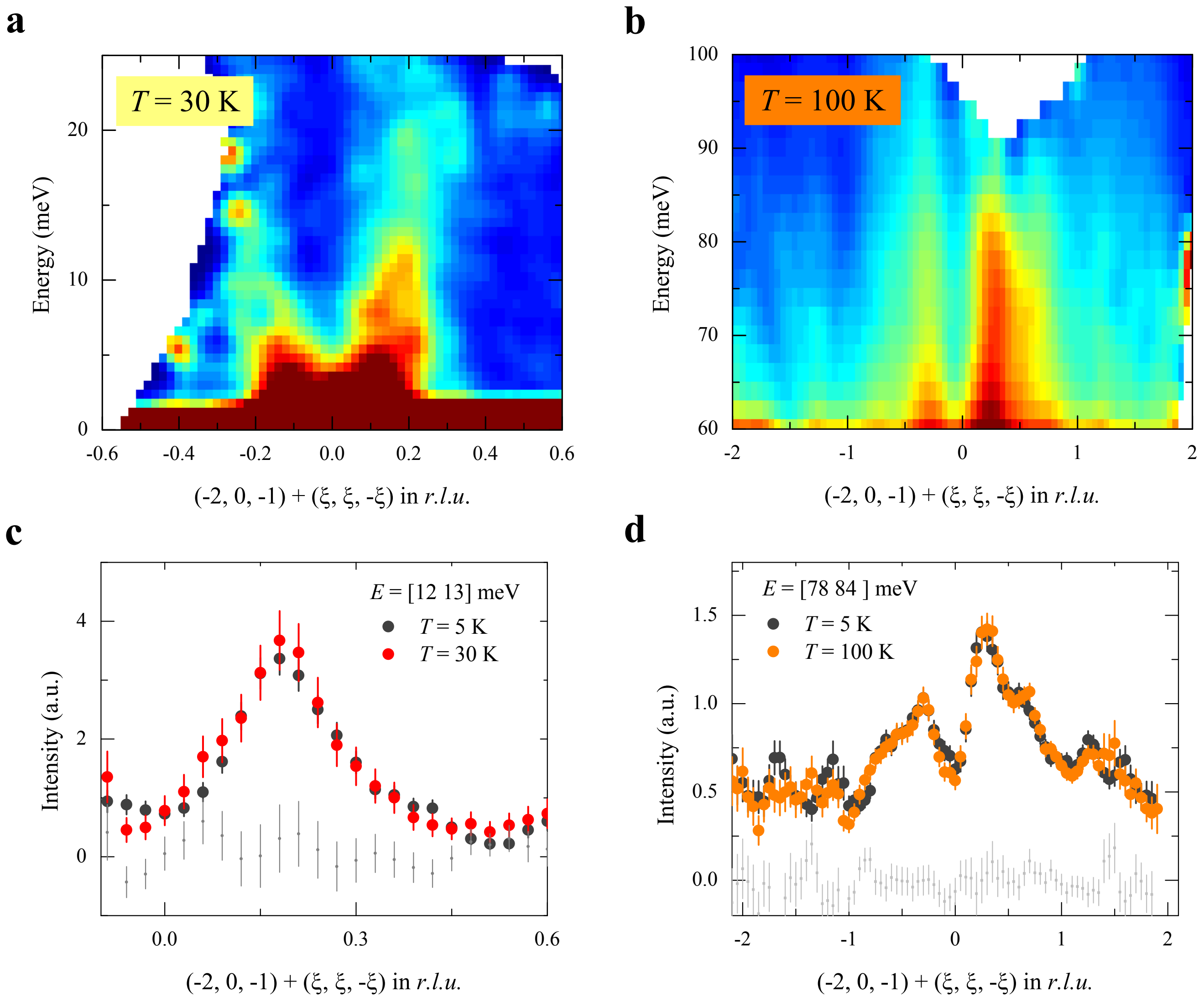

Supplementary Figure 5. Magnetic excitations at $T = 30$ and 100 K. (**a**-**b**) The E-$\mathbf{Q}$ slices along $(\xi, \xi, -\xi)$ direction around the $(-2, 0, -1)$ zone center at $T = 30$ K and 100 K, respectively. (**c**-**d**) Corresponding constant-E cuts at 12.5 meV and 81 meV, respectively. The subtraction of peak intensities between high and low temperatures are displayed by scattered gray symbols. Error bars represent one standard deviation of the data.

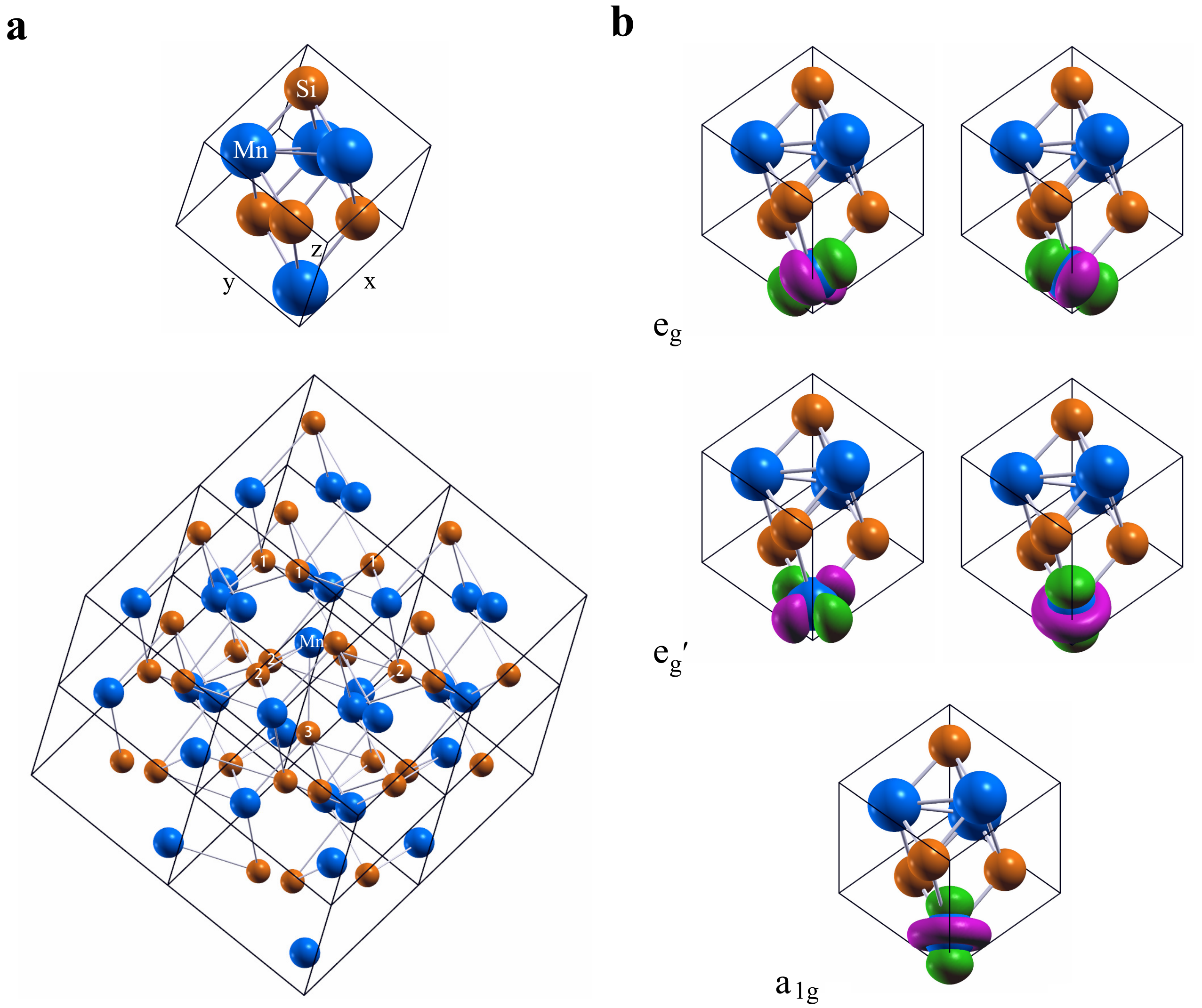

Supplementary Figure 6. (Color online) Structural and orbital data for MnSi. (**a**) B20 crystal structure of MnSi. Top: Cubic unit cell (lattice constant $a = 4.558$ Å) with four Mn sites (blue) and four Si sites (orange). Bottom: 2×2×2 supercell displaying the seven nearest-neighbor Si sites, split into 3 classes. (**b**) Projected-local orbitals of Mn($3d$) kind: e_g (top), $e_g{}'$ (middle) and a_{1g} (bottom).

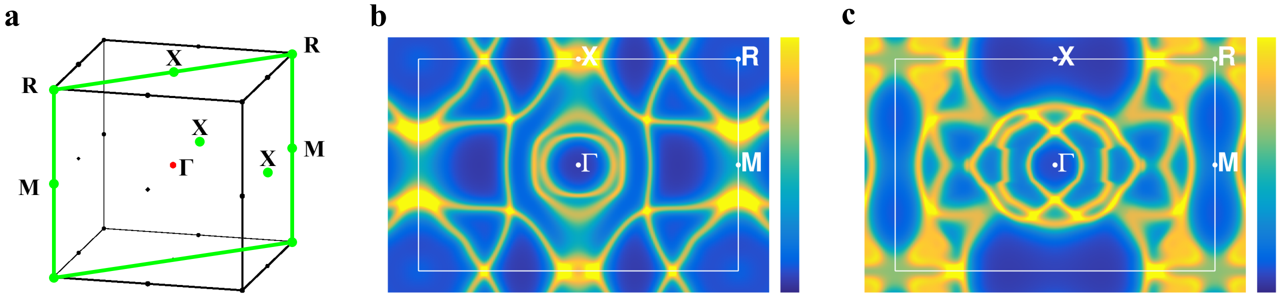

Supplementary Figure 7. (Color online) Fermiology of MnSi. (**a**) Brillouin zone with high-symmetry points and chosen plane (green) for two-dimensional cuts of the Fermi surface shown in (**b**,**c**). (**b**) DFT Fermi surface and (**c**) DFT+DMFT Fermi surface ($U = 2\,\mathrm{eV}$ and $J_\mathrm{H} = 0.65\,\mathrm{eV}$).

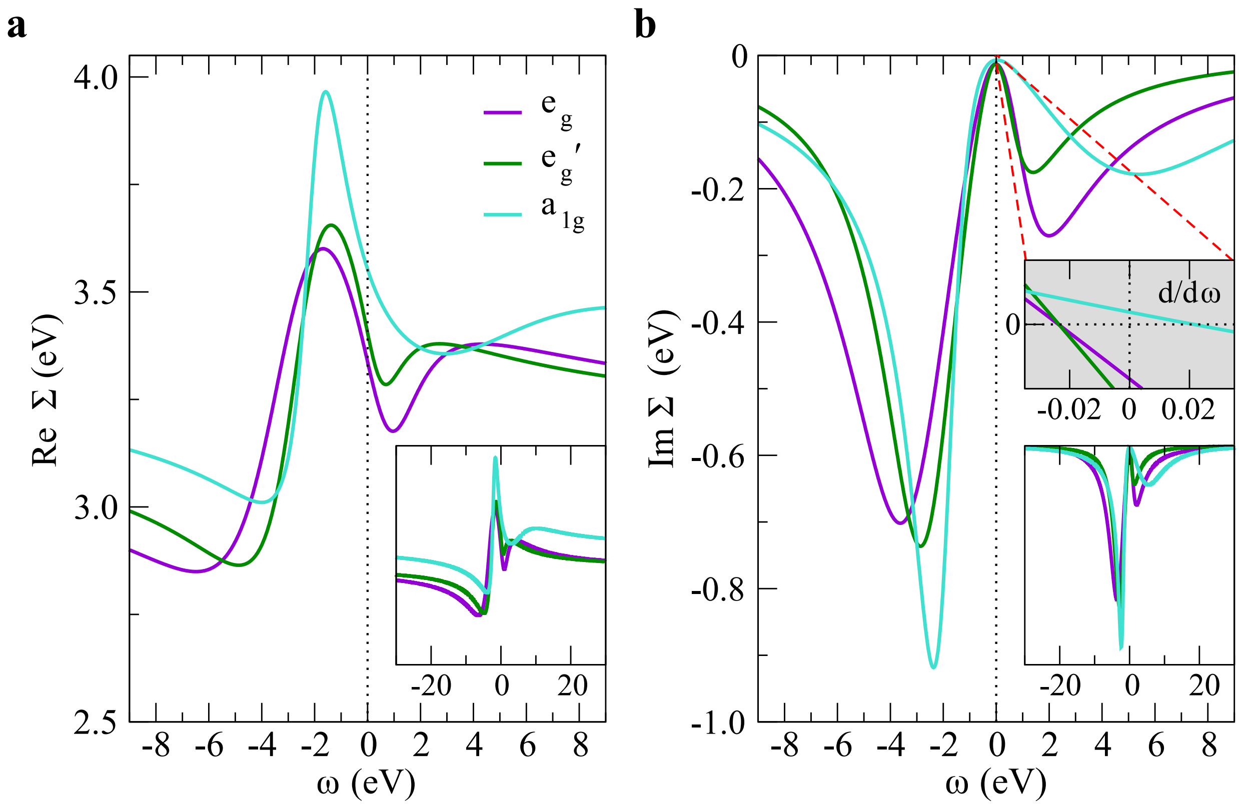

Supplementary Figure 8. (Color online) The orbital-resolved Mn self-energy within DFT+DMFT on the real-frequency axis ($U = 2\,\mathrm{eV}$ and $J_{\mathrm{H}} = 0.65\,\mathrm{eV}$). (**a**) Real part of the self-energy and (**b**) imaginary part of the self-energy. Larger insets provide the data in a wide-frequency window. The smaller grey inset in panel (**b**) part shows $\frac{d}{d\omega}\,\mathrm{Im}\,\Sigma$ about $\omega = 0$.

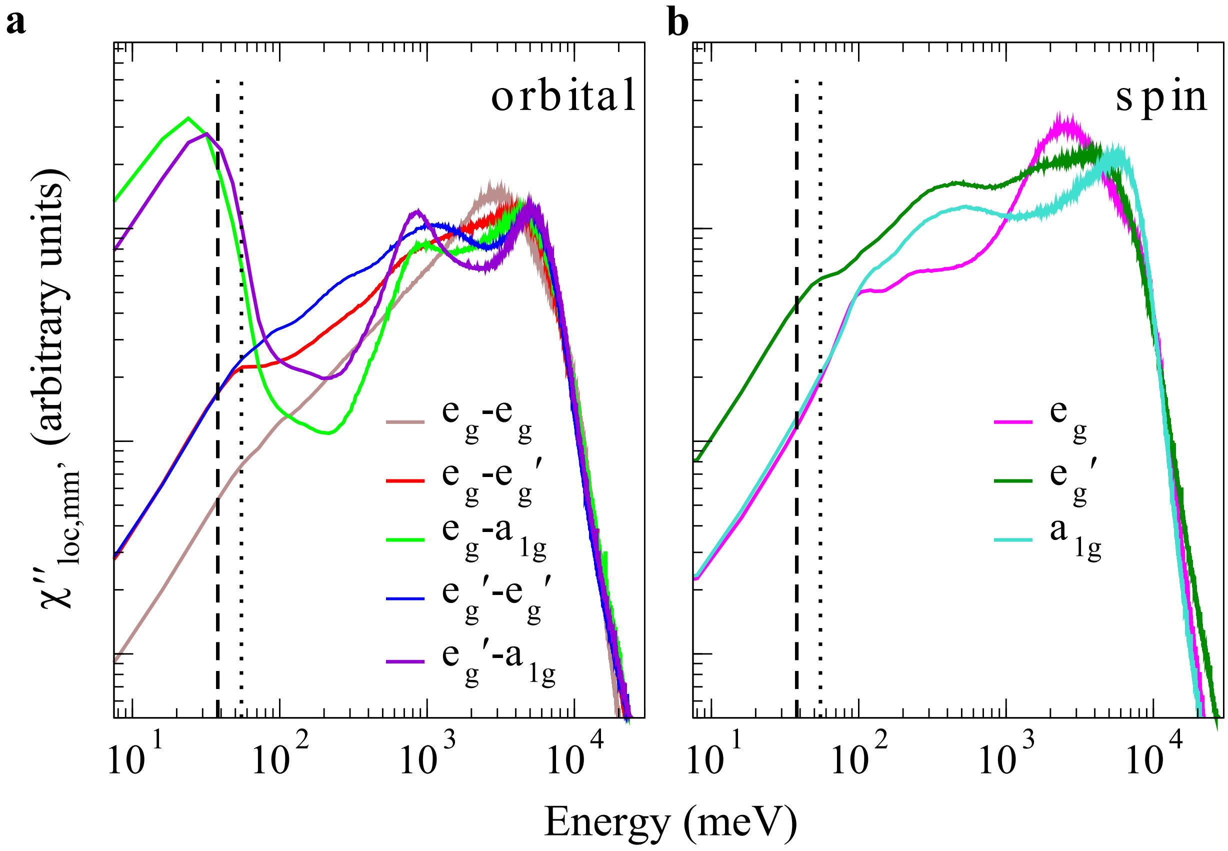

Supplementary Figure 9. (Color online) Orbital-resolved contributions to the local susceptibility in the (**a**) orbital and (**b**) spin sectors at $T = 300\,\mathrm{K}$ ($U = 2\,\mathrm{eV}$ and $J_{\mathrm{H}} = 0.65\,\mathrm{eV}$). Vertical lines mark the orbital (dashed) and spin (dotted) coherence scales as revealed from the total local susceptibilities (see main text). Note that for the local spin susceptibility, only the orbital-diagonal terms are shown.

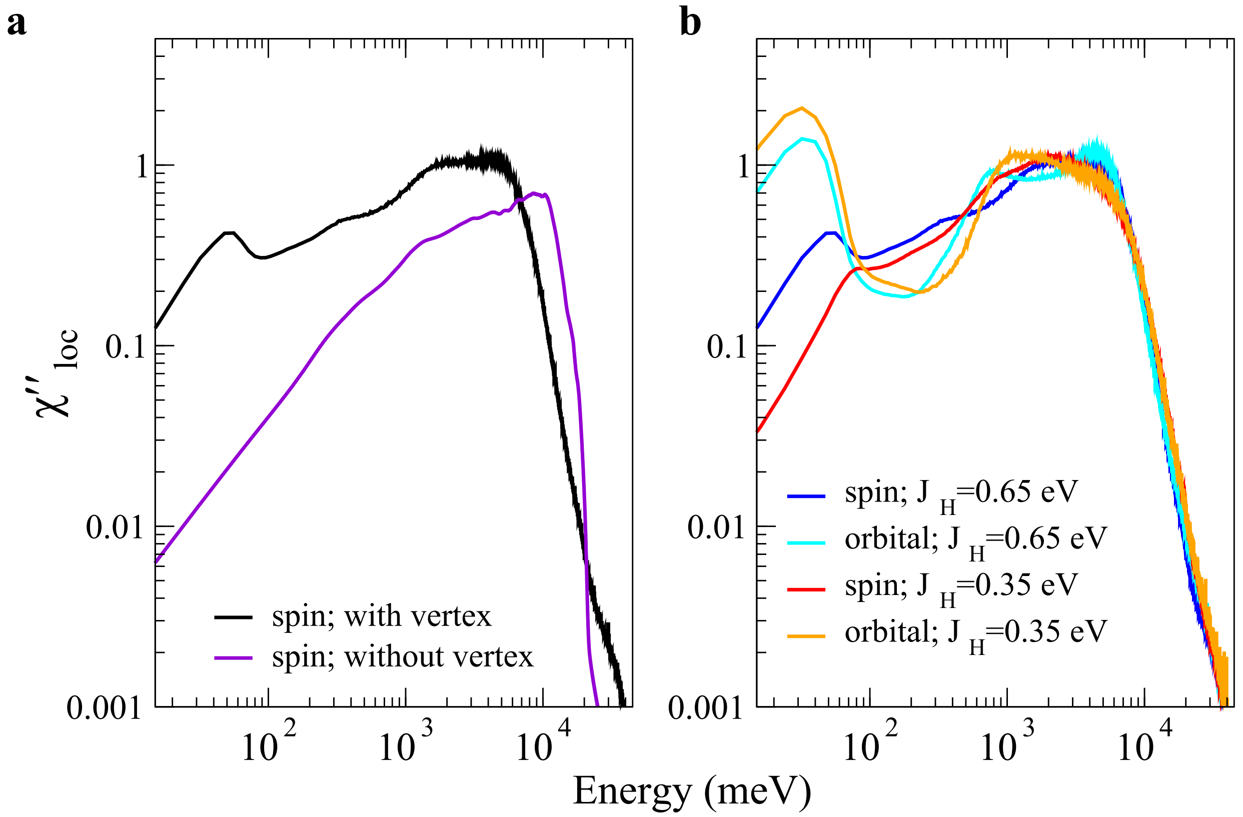

Supplementary Figure 10. (Color online) Variations of the imaginary part of the local susceptibility. (**a**) Full function (black) compared to the sole bubble part (violet), described by eq. (10), for the spin flavor ($U = 2\,\text{eV}$ and $J_{\text{H}} = 0.65\,\text{eV}$). (**b**) Comparison of orbital and spin functions for $U = 2\,\text{eV}$ and different J_{H}.

Supplementary Note 1: Parameterization of spin excitations

The dispersion relations for spin excitations shown in the main text's Fig. 2 are obtained by constant energy cuts at momentum positions chosen along high symmetry directions. As the spin wave excitations are highly damped (broad in E) and have a steep dispersion, constant E cuts were deemed to the most reliable way to extract this information. Supplementary Figure 1 (**a**) shows one representative energy-momentum (E -**Q**) slice with both damped wave excitations and weak acoustic phonon contributions. One representative line cut is shown in Supplementary Figure 1(**b**) highlighting these two contributions. The data focuses about the $\mathbf{Q} = (1, 1, 0)$ zone center along the (ξ, ξ, 0) direction, chosen due to the fact that the acoustic phonon contribution is very weak and the magnetic contribution is dominant.

Supplementary Figure 1 (**a**) presents the E - **Q** slice along the (ξ, ξ, 0) direction with a momentum step size of $\delta q_{||}$ = 0.02 r.l.u., out-of-plane momentum integration of $\delta q_{\perp}$=0.06 r.l.u. in both (0, 0, ξ) and ($-\xi$, ξ, 0), and an energy step size δE = 0.6 meV. Here $\delta q_{||}$ defines the momentum step size along the chosen **Q** direction in r.l.u., while $2\delta q_{\perp}$ defines the extent of data integration perpendicular to the **Q** direction—for instance the data is integrated along (0, 0, ξ) from -0.06 to 0.06 r.l.u.. Supplementary Figure 1 (**b**) shows a corresponding constant-energy cut integrated through δE from 5.4 to 6 meV, which is then fit by two pairs of Gaussians symmetrically displaced from the zone center (with unique widths for each pair) plus a constant background term. The overall fit is represented by two damped spin wave peaks (shaded green peaks) and two acoustic phonon peaks (shaded pink peaks).

The damped spin wave peaks show full width at half maxima (FWHM) of 0.14 r.l.u. (horizontal green bar) and are much broader than the phonon peak widths (horizontal pink bar, FWHM = 0.06 r.l.u.). We note that the intensity differences of light-green magnetic peaks arises due to the magnetic form factor in uncorrected data. The longitudinal acoustic phonon peak positions fit from cuts along the (ξ, ξ, 0) direction are overplotted in Supplementary Figure 1 (**a**) as pink circles, and the olive circles represent the low energy damped spin wave peak positions extracted from fits. This procedure was repeated in other zones and along other high symmetry directions at different incident energies to obtain the dispersion of magnetic excitations presented in Fig. 2 of the main text.

Acoustic phonon contributions to the scattering weights along high symmetry directions were verified via comparisons across multiple zones and in higher zones where the spin contributions vanish, and the dispersion observed agrees with published data in Lamago et al. [1]. The influence of higher energy optical phonons was also characterized and is illustrated in representative (E - **Q**) slices within Supplementary Figure 2. While phonon contributions to the scattering cross section prevent unambiguous identification of magnetic excitations across much of the energy window between 20 to 60 meV, there remains one small window in the energy range δE = [45, 50] meV where magnetic excitations can be isolated and analyzed. At higher energies above the single phonon cutoff energy $E \approx 60$ meV, characterization of magnetic excitations can be resumed free of interference from coherent phonons. Supplementary Figure 3 illustrates magnetic excitations above 60 meV across different zones and along varying high symmetry directions. Prominent chimney-like excitations appear away from the zone-boundary positions in momentum space, and their magnetic nature is further corroborated by the general trend of decreasing intensities at large $|\mathbf{Q}|$ positions.

The damping constants γ parameterizing the damped spin wave modes in the continuum discussed in the main text are obtained by performing constant-**Q** cuts at select positions. Supplementary Figure 4 shows a representative procedure with incident energy E_{i} = 30 meV at T = 5 K. The (-2, 0, -1) zone is selected because of minimal phonon contributions, and a typical E - **Q** slice is shown in Supplementary Figure 4 (**a**) (dE = 1.0 meV, $dq_{||}$ = 0.03 r.l.u., $dq_{\perp}$ = 0.075 r.l.u.). Cuts through this data were then fit to a damped Lorentzian with the form $I(E) \propto \frac{\gamma E}{(E^2-E_0^2)^2+(\gamma E)^2}$. For instance, fitting data at $\mathbf{Q}$ = (-1.82, -0.18, -1.18) r.l.u. in Supplementary Figure 4 (**b**), the resulting damping constant $\gamma = 19.2 \pm 6.5$ meV is 12 times the instrumental energy resolution of 1.6(1) meV (horizontal blue bar) at the elastic ($E = 0$) position. This procedure was repeated along other high symmetry directions to obtain the data presented in the main text's Fig. 2(**d**).

Supplementary Note 2: Gapped mode in weak ferromagnetic metal

Following the Laudau kinetic equation firstly used by Abrikosov and Dzyaloshinksii for weak ferromagnetic metal [2], in the low temperature regime where the spin dynamics are collisionless and dominated by spin precession, a new gapped mode induced by collective oscillations of the spin current is predicted to propagate outside the particle-hole continuum [3]. Without considering the influence of the Stoner continuum first, the dispersion is predicted for weak ferromagnetic metals with Landau parameters f_0^{a}, f_1^{a} and magnetization m [4]:

$$E^2 + 2m(f_0^{\rm a} - \frac{f_1^{\rm a}}{3})E + c_{\rm s}^2 q^2 = 0 \tag{1}$$

where $c_{\rm s}^2 = |1 + f_0^{\rm a}|(1 + f_1^{\rm a}/3)v_{\rm F}^2/3$ is the spin wave velocity and $v_{\rm F}$ the Fermi velocity. Rewriting the equation in terms of the gap size Δ of the gapped mode at $q = 0$ and spin wave stiffness constant D from the gapless Goldstone mode, we have

$$E^2 - \Delta E + (D \cdot \Delta)q^2 = 0 \tag{2}$$

where $\Delta = -2m(f_0^{\rm a} - \frac{f_1^{\rm a}}{3})$ and $D = c_{\rm s}^2/\Delta$. Thus, in the small q limit, the spin wave ($E_{\rm s}$) and Δ modes (E_Δ) disperse as:

$$E_{\rm s} = Dq^2 \tag{3}$$

$$E_\Delta = \Delta - Dq^2 \tag{4}$$

In the small q limit, the two modes then follow the general equations

$$E_{\rm s} = \frac{\Delta - \sqrt{\Delta^2 - 4D\Delta q^2}}{2} \tag{5}$$

$$E_\Delta = \frac{\Delta + \sqrt{\Delta^2 - 4D\Delta q^2}}{2} \tag{6}$$

Equation 5 is applied to fit the experimentally observed spin wave dispersion, where a small anisotropy along different high symmetry directions is observed as shown in Fig. 2 (**b**) of the main text. The fits are surprisingly good for q up to $0.5\mathring{A}^{-1}$ along the $(1, 1, 0)$ direction with fitting parameters $\Delta = 48$ meV and $D \approx 46$ meV, where D is very close to reported value in [5].

Notice that equations 5 and 6 are only valid when $\Delta^2 - 4D\Delta q^2 > 0$, or only when q follows

$$q < q_{\rm s} = \frac{1}{2}\sqrt{\frac{\Delta}{D}} \tag{7}$$

In this simple model, the spin wave and Δ-mode will merge at the special point $q_{\rm s} = \frac{1}{2}\sqrt{\frac{\Delta}{D}}$, as depicted in Fig. 2(**c**) in the main text. Upon approaching $q_{\rm s}$ from below, the spin wave velocity rapidly increases, and diverges at $q = q_{\rm s}$. When $q > q_{\rm s}$, the equation 2 indicates the non-propagating nature of the damped spin wave mode. For better comparison with experiment and damping from the Stoner continuum, we further simulate the spin wave damping by including the empirically determined, linearly increasing, damping parameter ($\gamma \sim E$) along with the experimentally determined q and E dependent intensities in Fig. 2(**c**) of the main text.

Supplementary Note 3: Temperature dependence of spin excitations

Magnetic excitations observed in the ordered state ($T = 5$ K) can be compared with data at higher temperatures in the paramagnetic state, as illustrated in Supplementary Figure 5. The data indicates no observable changes in the magnetic spectrum upon leaving the ordered state. To help show this, the E-$\mathbf{Q}$ slices along the (ξ, ξ, $-\xi$) direction around (-2, 0, -1) zone at $T = 30$ K and 100 K are displayed in Supplementary Figure 5 (**a-b**), respectively. The high temperature E-$\mathbf{Q}$ slices can be directly compared to low temperature slices with the same configurations as Fig. 1 of the main text and in Supplementary Figure 3. For better comparison, constant-E cuts are performed and compared in Supplementary Figure 5 (**c-d**). Negligible differences in peak intensities are observed upon warming as illustrated by direct subtraction of the data shown as light gray symbols.

Supplementary Note 4: MnSi crystal structure and effective Mn(3d) orbitals

The MnSi compound crystallizes in the noncentrosymmetric B20 structure (space group $P2_13$) with four Mn and four Si atoms in the cubic unit cell (see Supplementary Figure 6 (**a**, top). Though belonging to the cubic crystal system, the local environment amounts to trigonal coordination of the atoms in (111) direction, rendering the crystal structure and the resulting electronic structure intriguing. Each Mn site is surrounded by seven nearest-neighbor Si sites that split into three different classes upon the Mn-Si distance (cf. Supplementary Figure 6 (**a**, bottom) : three atoms of class Si1 with largest distance, three atoms of class Si2 with intermediate distance and one atom forming class Si3 with shortest distance.

Because of the strong hybridization between Mn and Si, projected-local orbitals for Mn(3d) are constructed based on forty Kohn-Sham states, starting from the bottom of the Kohn-Sham spectrum. The local projection functions consist of the five linear combinations of atomic 3d orbitals that diagonalize the Mn(3d) orbital-density matrix. Supplementary Figure 6 (**b**) displays the set of projected-local orbitals on the DFT level, *i.e.* twofold-degenerate e_g, twofold-degenerate ${e_{\rm g}}'$ as well as $a_{\rm 1g}$. The three different orbital classes may also be categorized according to their character in view of pointing to the environment. The $e_{\rm g}$ orbitals favorably point to near-neighbor Mn sites, the ${e_{\rm g}}'$ orbitals are more or less equally balanced in pointing to Mn and Si, and the $a_{\rm 1g}$ orbital is favorably pointing to Si, *i.e.* one lobe of $a_{\rm 1g}$ is directly oriented toward the Si3 class.

The DFT crystal-field level energies for these effective Mn(3d) orbitals read $\{\varepsilon_{e_{\rm g}}, \varepsilon_{{e_{\rm g}}'}, \varepsilon_{a_{\rm 1g}}\} = \{328, 383, 442\}$ meV. But due to the strong dispersion effects in this large-bandwidth material, the DFT+DMFT orbital occupations for $U = 2$ eV and $J_{\rm H} = 0.65$ eV at $T = 300$ K amount to $\{N_{e_{\rm g}}, N_{{e_{\rm g}}'}, N_{a_{\rm 1g}}\} = \{1.22, 1.27, 1.40\}$ per individual orbital. All orbitals contribute to the multi-sheet Fermi surface, the system represents an example of a distinct five-orbital correlated material.

Supplementary Note 5: MnSi Fermiology and DFT+DMFT self energies

A two-dimensional cut (see Supplementary Figure 7 (**a**) of the MnSi Fermi surface is shown within DFT in Supplementary Figure 7 (**b**) and within DFT+DMFT in Supplementary Figure 7 (**c**). The strong differences as noted in the main text are easily observable.

The orbital-resolved Mn self-energy on the real-frequency axis is displayed in Supplementary Figure 8 with its real and imaginary parts. For the imaginary part, one finds that there is small difference between the low-energy maximum of $\mathrm{Im}\,\Sigma$ for $\{e_{\rm g}, {e_{\rm g}}'\}$ and $a_{\rm 1g}$. This becomes best obvious when computing $\frac{d}{d\omega}\,\mathrm{Im}\,\Sigma$ and looking for the zeros (see small grey inset in Supplementary Figure 8 (**b**). While $e_{\rm g}$ and ${e_{\rm g}}'$ have identical maxima at $\omega_{\rm max} = -0.023$ eV, the one for $a_{\rm 1g}$ reads $\omega_{\rm max} = 0.021$ eV. The energy difference $\Delta_{\rm max} = 44$ meV roughly matches the low-energy coherence scale of the local orbital susceptibility, discussed in the main text.

Supplementary Note 6: Additional analysis of MnSi local susceptibilities

Orbital-resolved Mn susceptibilities

The local susceptibility $\chi_{\rm loc}$ is a two-particle function which includes a so-called 'bubble' term, described by the convolution of two one-particle Green's functions, as well as a manifest two-particle contribution, the so-called 'vertex corrections'. As function of imaginary time τ, the local susceptibility $\chi_{\rm loc}$ of orbital and of spin kind may be written as

$$\chi^{(\rm o)}_{\rm loc}(\tau) = \sum_{mm'} \chi^{(\rm o)}_{{\rm loc},mm'}(\tau) = \sum_{mm'} \left\langle \mathcal{T}_\tau \left[\frac{1}{2}(n_m(\tau) - n_{m'}(\tau))\right] \left[\frac{1}{2}(n_m(0) - n_{m'}(0))\right] \right\rangle \quad , \tag{8}$$

$$\chi^{(\rm s)}_{\rm loc}(\tau) = \sum_{mm'} \chi^{(\rm s)}_{{\rm loc},mm'}(\tau) = g^2 \sum_{mm'} \left\langle \mathcal{T}_\tau \left[\frac{1}{2}(n_{m,\uparrow}(\tau) - n_{m,\downarrow}(\tau))\right] \left[\frac{1}{2}(n_{m',\uparrow}(0) - n_{m',\downarrow}(0))\right] \right\rangle \quad , \tag{9}$$

where m, m' mark orbitals, $n_{m\sigma} = c^\dagger_{m\sigma} c_{m\sigma}$ ($\sigma =\uparrow,\downarrow$), $n_m = n_{m,\uparrow} + n_{m,\downarrow}$ and $g = 2$.

Supplementary Figure 9 displays the imaginary-part components $\chi''_{{\rm loc},mm'}$ of the local Mn susceptibilities. In case of the orbital susceptibility, the dominant contribution of fluctuations contributing to the peak structure below 100 meV are the ones into the $a_{\rm 1g}$ orbital from $\{e_{\rm g}, {e_{\rm g}}'\}$. This finding is in line with the energy scale $\Delta_{\rm max}$ (see last section) being responsible for the low-energy coherence peak in the local orbital susceptibility. While the intra $e_{\rm g} - e_{\rm g}$ fluctuations seemingly do not play a significant role, the intra ${e_{\rm g}}' - {e_{\rm g}}'$ and inter $e_{\rm g} - {e_{\rm g}}'$ fluctuations are still considerable. For the spin sector, we exhibit only the orbital-diagonal terms, but note that the total spin susceptibility consists also of orbital off-diagonal terms. Those should not be disregarded in order to obtain the complete peak-feature structures at lower energies. Apparently, the ${e_{\rm g}}'$ spin-fluctuation contribution is strongest in the relevant coherence-scale energy regime below 100 meV.

Since the ${e_{\rm g}}'$ orbital marks prominent contributions for both coherence-scale formations, i.e. orbital and spin, its role in the coupling of orbital and spin fluctuations should be decisive. Yet it is also obvious from the data that the whole structure of orbital and spin susceptibilities is manifestly multi-orbital, in character *i.e.* there is not a single orbital out of the manifold that dominates the physics.

Influence of vertex corrections and Hund's coupling $J_{\rm H}$

The vertex corrections are essential for the physics of the MnSi low-energy coherence scales in the local susceptibility. This is shown in Supplementary Figure 10 (**a**), where we plot the imaginary part of the full local spin susceptibility (i.e. with including vertex corrections) along with the sole bubble part. The latter one is obtained from the DFT+DMFT total spectral function $A(\omega)$ via

$$\mathrm{Im}\,\chi_{\rm loc}(\omega) \sim \int d\omega'\, A(\omega') A(\omega' + \omega)\, \left[f(\omega') - f(\omega' + \omega)\right] \quad . \tag{10}$$

Here, $f(y) = \frac{1}{{\rm e}^{\beta y}+1}$ is the Fermi-Dirac distribution function with the inverse temperature β. While the part of the intermediate-energy and high-energy structure of $\mathrm{Im}\,\chi_{\rm loc}$ is reproduced by this simplified description, the low-energy coherence structure below 100 meV is completely absent.

The Supplementary Figure 10 (**b**) exhibits the imaginary part of the local spin and orbital susceptibility for $U = 2$ eV and two different $J_{\rm H}$ values. It is seen that the low-energy coherence spin and orbital scales split further with smaller Hund $J_{\rm H}$. On the other hand, the intermediate (pre-)scales, visible by the "humps" between 0.1 and 1 eV, appear to shift up in energy and also come closer for $J_{\rm H} = 0.35$ eV. This is in line with the interpretation that the intermediate scales correspond to the "conventional" Hund-metal scenario, whereas the low-energy scales mark the "unconventional" scenario detected in the present case.

[1] Lamago, D. *et al.* Lattice dynamics in the itinerant helical magnet MnSi. *Phys. Rev. B* **82**, 144307 (2010).
[2] Abrikosov, A. & Dzialoshinskii, I. Spin waves in a ferromagnetic metal. *J. Exptl. Theoret. Phys.* **35**, 535–537 (1959).
[3] Bedell, K. S. & Blagoev, K. B. Quantum spin hydrodynamics and a new spin-current mode in ferromagnetic metals. *Philosophical Magazine Letters* **81**, 511–517 (2001).
[4] Zhang, Y., Farinas, P. & Bedell, K. The "Higgs" Amplitude Mode in Weak Ferromagnetic Metals. *Acta Physica Polonica A* **127**, 153–156 (2015).
[5] Ishikawa, Y., Shirane, G., Tarvin, J. A. & Kohgi, M. Magnetic excitations in the weak itinerant ferromagnet MnSi. *Phys. Rev. B* **16**, 4956–4970 (1977).
